# Detection of Extended-Spectrum Beta-Lactamase (ESBL)-Producing *Enterobacteriaceae* from Diseased Broiler Chickens in Lusaka District, Zambia

**DOI:** 10.3390/antibiotics13030259

**Published:** 2024-03-15

**Authors:** Chikwanda Chileshe, Misheck Shawa, Nelson Phiri, Joseph Ndebe, Cynthia Sipho Khumalo, Chie Nakajima, Masahiro Kajihara, Hideaki Higashi, Hirofumi Sawa, Yasuhiko Suzuki, Walter Muleya, Bernard Mudenda Hang’ombe

**Affiliations:** 1 Department of Biomedical Sciences, School of Veterinary Medicine, University of Zambia, Lusaka 10101, Zambiamuleyawalter@gmail.com (W.M.); 2 Hokudai Center for Zoonosis Control in Zambia, University of Zambia, Lusaka 10101, Zambia; misheckshawa@ymail.com (M.S.); kajihara@czc.hokudai.ac.jp (M.K.); h-sawa@ivred.hokudai.ac.jp (H.S.); 3 Department of Medicine Control, Zambia Medicines Regulatory Authority, Lusaka 10101, Zambia; nphiri39@yahoo.com; 4 Department of Disease Control, School of Veterinary Medicine, University of Zambia, Lusaka 10101, Zambia; j.ndebe@yahoo.com; 5 Division of Bioresources, International Institute for Zoonosis Control, Hokkaido University, N20 W10, Kita-ku, Sapporo 001-0020, Japansuzuki@czc.hokudai.ac.jp (Y.S.); 6 Division of International Research Promotion, International Institute for Zoonosis Control, Hokkaido University, N20 W10, Kita-ku, Sapporo 001-0020, Japan; 7 Division of Infection and Immunity, International Institute for Zoonosis Control, Hokkaido University, N20 W10, Kita-ku, Sapporo 001-0020, Japan; hidea-hi@czc.hokudai.ac.jp; 8 Institute for Vaccine Research and Development (HU-IVReD), Hokkaido University, N21 W11, Kita-ku, Sapporo 001-0020, Japan; 9 Division of Molecular Pathobiology, International Institute for Zoonosis Control, Hokkaido University, N20 W10, Kita-ku, Sapporo 001-0020, Japan; 10 Department of Para-Clinical Studies, School of Veterinary Medicine, University of Zambia, Lusaka 10101, Zambia; 11 Africa Centre of Excellence for Infectious Diseases of Humans and Animals, School of Veterinary Medicine, University of Zambia, Lusaka 10101, Zambia

**Keywords:** *Enterobacteriaceae*, *bla*_CTX-M_, *bla*_TEM_

## Abstract

Poultry products in Zambia form an integral part of the human diet in many households, as they are cheap and easy to produce. The burden of poultry diseases has, however, remained a major challenge. Growing consumer demand for poultry products in Zambia has resulted in non-prudent antimicrobial use on farms, intending to prevent and treat poultry diseases for growth optimisation and maximising profits. This cross-sectional study aimed to identify the different types of bacteria causing diseases in chickens in Lusaka and to detect the extended-spectrum lactamase (ESBL)-encoding genes. We collected 215 samples from 91 diseased chickens at three post-mortem facilities and screened them for Gram-negative bacteria. Of these samples, 103 tested positive for various clinically relevant *Enterobacteriaceae*, including *Enterobacter* (43/103, 41.7%), *Escherichia coli* (20/103, 19.4%), *Salmonella* (10/103, 9.7%), and *Shigella* (8/103, 7.8%). Other isolated bacteria included *Yersinia*, *Morganella*, *Proteus*, and *Klebsiella*, which accounted for 21.4%. *E. coli*, *Enterobacter*, *Salmonella,* and *Shigella* were subjected to antimicrobial susceptibility testing. The results revealed that *E. coli*, *Enterobacter*, and *Shigella* were highly resistant to tetracycline, ampicillin, amoxicillin, and trimethoprim-sulfamethoxazole, while *Salmonella* showed complete susceptibility to all tested antibiotics. The observed resistance patterns correlated with antimicrobial usage estimated from sales data from a large-scale wholesale and retail company. Six (6/14, 42.9%) *E. coli* isolates tested positive for *bla*_CTX-M_, whilst eight (8/14, 57.1%) *Enterobacter* samples tested positive for *bla*_TEM_. Interestingly, four (4/6, 66.7%) of the *E. coli* isolates carrying *bla*_CTX-M_-positive strains were also positive for *bla*_TEM_. Sanger sequencing of the PCR products revealed that five (5/6, 83.3%) of the abovementioned isolates possessed the *bla*_CTX-M-15_ allele. The results suggest the presence of potentially pathogenic ESBL-producing *Enterobacteriaceae* in poultry, threatening public health.

## 1. Introduction

Poultry production in Zambia is one of the most important activities in the livestock sector. The chicken population is estimated to be 94 million broilers, 15 million village chickens, and 5.8 million layers [1]. Moreover, poultry products in Zambia, like in other developing African countries, form an integral part of the human diet in many households, as they are cheaper source of animal protein and are easier to produce compared with other foods of animal origin [2,3].

The Zambian population is progressively expanding; similarly, the demand for meat and other foods increases, leading to food security problems [1]. As a result, the government relies on agricultural industries to heighten animal production and address food insecurity. These industries usually raise large numbers of animals by boosting production through extensive farming methods that involve antimicrobial growth promoters [4]. Although such livestock intensification approaches are essential for alleviating food shortages, they are also associated with the emergence and spread of antimicrobial resistance (AMR) [5]. This is further exacerbated by poorly upheld animal husbandry practices that result in frequent infections requiring antimicrobial use, leading to AMR. In addition, farmers sometimes deliberately underdose their livestock because of the high cost associated with antibiotics, worsening the problem.

AMR can be defined as the inability of bacteria parasites and viruses to respond to medicines, making infection treatment difficult [6]. AMR may be encoded chromosomally, but the production of plasmid-mediated extended-spectrum lactamases (ESBLs) is more common. There are nine ESBL classes, but common ones include variants of the CTX-M-type and derivatives of SHV-1, TEM-1, and TEM-2 [7]. ESBL-producing *Enterobacteriaceae*, resistant to third-generation cephalosporins such as cefotaxime (CTX), are dreaded profoundly because of their extensive geographic distribution and adverse health impacts. While ESBLs are more prevalent among hospital isolates, poultry has emerged as an important reservoir for possible zoonotic transmission [7]. This reservoir includes ESBL-encoding genes harboured by commensal and pathogenic strains and may disseminate to humans via two main mechanisms. Firstly, ESBL genes may be transmitted by horizontal gene transfer, and the treatment implications depend on the pathogenicity of the recipient bacterial strain [8]. Of greater concern is the direct transmission of disease-causing pathogens by clonal expansion, potentially leading to clinical disease and treatment failure. Therefore, understanding the zoonotic transmission of poultry-associated ESBLs requires a multipronged approach that considers both healthy and diseased chickens. In Zambia, most studies have focused on non-pathogenic bacteria isolated from asymptomatic chickens [2,9,10], leaving a gap in the ESBL status among sick chickens. Thus, this study was undertaken to estimate antimicrobial usage, isolate bacterial pathogens, and confirm the presence of ESBL-encoding genes by PCR and sequencing in bacteria isolated from poultry.

## 2. Results

### 2.1. Antibiotics Sales

**Figure 1:**
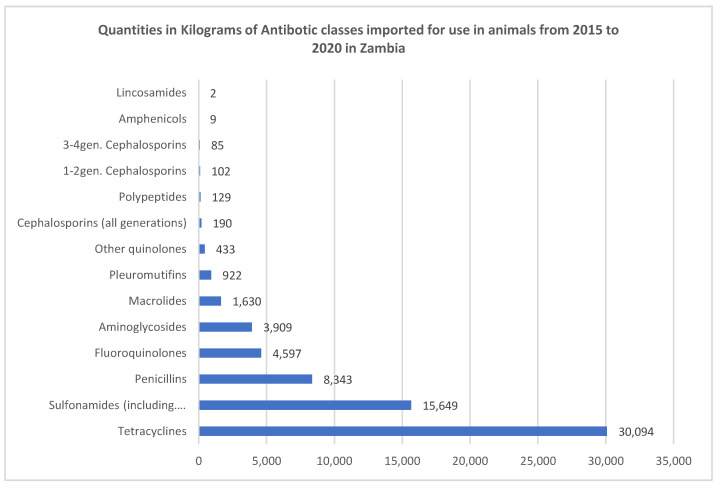
Importation of antibiotics from 2015 to 2020 [11].

**Figure 2:**
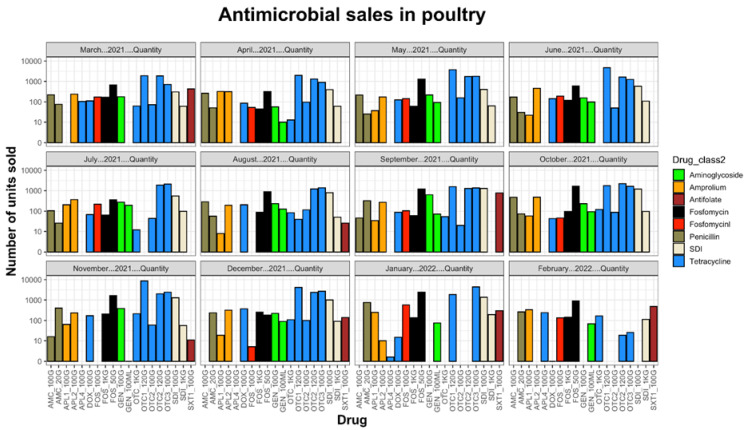
Sales data from March 2021 to February 2022.

Antibiotic importation data from 2015 to 2022 showed that tetracyclines were the most imported antibiotics, followed by sulphonamides, and then penicillins (Figure 1). The imported tetracyclines included doxycycline, oxytetracycline, and chlortetracycline. The high influx of tetracyclines was consistent with the inexpensive nature, broad-spectrum activity, and minimal side effects of this antibiotic class. The sulphonamides included sulfamethoxazole, and the sulfadiazine/trimethoprim combination. The high influx of sulphonamides could be attributed to their use in the treatment of coccidiosis and colibacillosis, which are among the most common poultry infections. Finally, the penicillin group included amoxicillin and ampicillin. The high importation levels of tetracyclines, sulphonamides, and penicillins coincide with sales data (Figure 2), which equally show high sales volumes of the named antimicrobials. Seventy percent (70%) of these antimicrobials are sold to Lusaka and Copperbelt provinces, whilst the remaining 20% is shared amongst the other provinces. The high antibiotic consumption in Lusaka and the Copperbelt is consistent with the observation that these two provinces have the highest number of poultry farms in the country coupled with high population densities [1].

All poultry antimicrobials sold were for oral administration following the presentation of a valid licence (for companies) or prescription (for farmers). Throughout the year, the trend in the sales had minimal fluctuation, with tetracyclines being the most sold antibiotics, followed by sulphonamides, penicillins, and aminoglycosides (Figure 2).

### 2.2. Prevalence of Enterobacteriaceae

In total, 215 samples were screened from 91 diseased chickens aged three to five weeks across Lusaka of which 103 (103/215, 43%) tested positive for potentially pathogenic Gram-negative bacteria, mostly belonging to the *Enterobacteriaceae* family. The major pathogenic species of clinical relevance identified were *Enterobacter* (43/103, 41.7%), *E. coli* (20/103, 19.4%), *Salmonella* (10/103, 9.7%), *Proteus* (10/103, 9.7%), and *Shigella* (8/103, 7.8%) (Table 1. Other isolated bacteria included *Yersinia*, *Morganella*, and *Klebsiella*, which accounted for 19.5% (Table 2).

### 2.3. Antimicrobial Sensitivity Showed Multidrug Resistance (MDR) among Enterobacteriaceae

**Figure 3:**
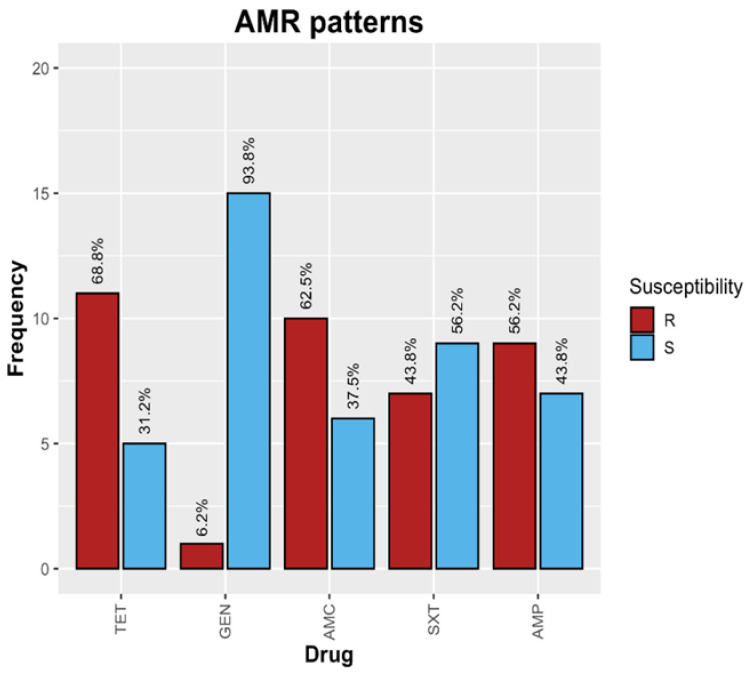
Overall AST for 16 *Enterobacteriaceae* strains.

**Figure 4:**
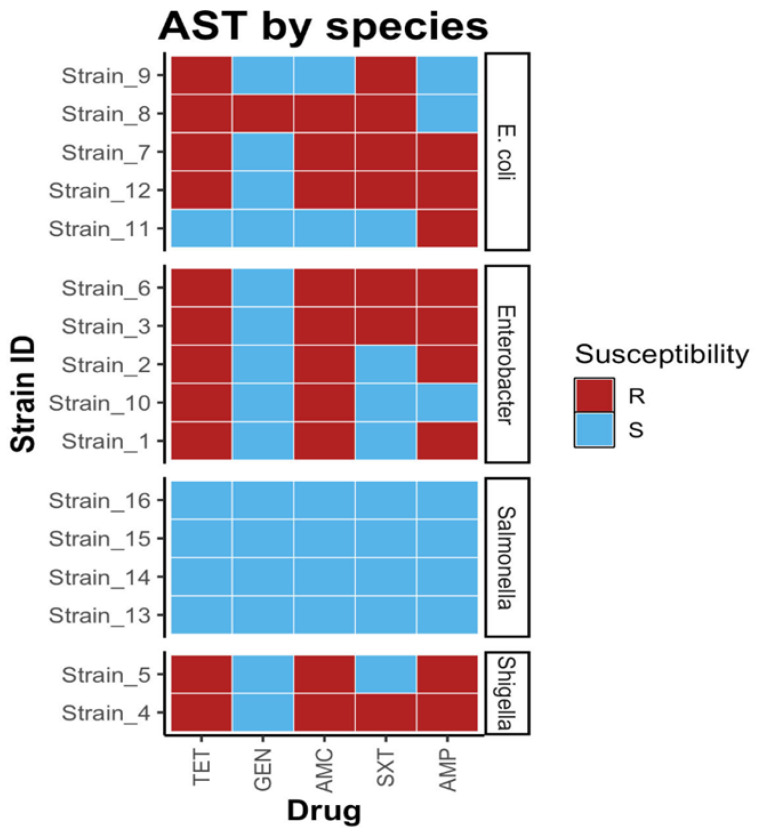
AMR patterns for the various bacteria.

From the identified *Enterobacteriaceae* strains (*n* = 103), 16 were randomly picked, including *Enterobacter* (*n* = 5), *E. coli* (*n* = 5), *Salmonella* (*n* = 4), and *Shigella* (*n* = 2). These strains were subjected to antibiotic sensitivity testing (AST) against five antibiotics; the results showed the highest resistance to tetracycline (11/16, 68.8%), followed by amoxicillin (10/16, 62.5%), ampicillin (9/16, 56.2%), cotrimoxazole (7/16, 43.8%), and gentamicin (1/16, 6.2%) (Figure 3). MDR was noted if the bacterial isolate was resistant to three or more groups of antibiotics [12]. In addition, MDR was observed in *E. coli* (3/5, 60%), *Enterobacter* (2/5, 40%), and *Shigella* (1/2, 50%), while *Salmonella* showed complete susceptibility to all antibiotics (Figure 4). By area sampling, most of the samples showing resistance were from post-mortem facility A (Table 2), which is centrally located compared with the other sites.

### 2.4. Association between Cefotaxime (CTX) Resistance and ESBL Genes

To quantify CTX resistance, the isolates were subjected to broth microdilution. The results indicated that 14 out of 103 (13.6%) isolates were CTX-resistant, of which 57.1% (8/14) were *Enterobacter* and 42.9% (6/14) were *E. coli*. Notably, one *Enterobacter* and three *E. coli* strains exhibited high levels of resistance with CTX MICs of at least 512 µg/mL (Table 3). In addition, all the *E. coli* isolates (*n* = 6) were *bla*_CTX-M_ gene-positive on PCR, and 66.7% (4/6) carried the *bla*_TEM_ gene. Furthermore, all eight *Enterobacter* isolates possessed only *bla*_TEM_. However, none of the 14 isolates harboured the genes *bla*_OXA_ and *bla*_SHV_. Sequence analysis of the six *E. coli bla*_CTX-M_-positive isolates showed the presence of the *bla*_CTX-M-15_ allele in all the isolates.

## 3. Discussion

The documentation of antimicrobial importation and sales data is vital as it serves as a basis for intervention programs and policy decisions. In this study, antibiotic disk selection was based on sales data from March 2021 to February 2022 from one of the country’s largest wholesale and retail outlets of animal pharmaceuticals. The most sold group of antibiotics was represented by tetracyclines, followed by penicillins, which corresponded to AST results which showed that 68.8% of the isolates were resistant to tetracyclines, 62.5% to amoxicillin, and 56.2% to ampicillin. The high levels of tetracycline and penicillin resistance observed in this study are similar to what has been reported previously [9]. This could be attributed to the huge quantities of tetracyclines and penicillins being imported and sold in the country, as reflected in the sales data (Figure 2). These results also concur with a study performed in Tanzania and Cameroon, where tetracyclines, penicillins, and sulfonamides were the most used antimicrobials in poultry production [13,14].

The cumulative rise in AMR could firstly be attributed to the use of antibiotics for infection control rather than treatment of disease by poultry farmers. This is usually conducted by introducing antibiotics in the first week of the chick’s life to counter infections that may rise due to breaches in biosecurity [15]. In Zambia, farmers avoid economic losses from infections by medicating healthy chickens up to the sixth week, despite being aware of the withdrawal periods [16]. Secondly, despite large agro shops dispensing antimicrobials by prescription, farmers still have access to antimicrobials in smaller outlets in the central business district [17]. Furthermore, the lack of knowledge by farmers is a key attribute contributing to the development of MDR, as some farmers are of the belief that the use of different antibiotics lowers the chances of AMR development [18].

Over the past few years, AMR among the *Enterobacteriaceae* family has skyrocketed worldwide [19]. Studies pertaining to AMR have frequently been reported in *E. coli* and *Salmonella* from healthy chickens [2,20]. In this study, the most predominant species isolated was *Enterobacter* (43/103, 47%), which showed multidrug resistance to tetracyclines, sulphonamides, ampicillin, and amoxicillin. Sulfonamide and penicillin resistance are commonly reported among *Enterobacteriaceae* in other studies [21], probably due to the overuse of these two drug classes in poultry. For instance, previous studies have revealed a corelation between tetracycline concentrations and resistance in the environment [22], as well as a relationship between tetracycline usage and resistance in poultry [23].

In our study, the prevalence of *E. coli* was 19.4% (20/103), which was lower than what has been reported elsewhere. For instance, Ibrahim et al. (2019) and Ameen-Ur-Rashid et al. (2016) found approximately 34% and 35% *E. coli* isolates in diseased chickens in Jordan and Pakistan, respectively [24,25]. Additionally, a higher percentage of *E. coli* (75.5%) isolates was observed in a study by Engy Ahmed Hamed et al. [26]. Despite the lower *E. coli* prevalence, 60% (3/5) of the tested *E. coli* isolates exhibited MDR, involving tetracyclines, penicillins, and suphomonamides. This observation could suggest the clonal expansion of one strain or the horizontal transfer of an MDR mobile element (e.g., a plasmid); therefore, detailed characterization by whole-genome sequencing will be required to confirm the hypothesis.

In total, 10 out of the 103 (9.7%) of the samples were *Salmonella*. A recent study in Copperbelt Province in Zambia reported a higher *Salmonella* prevalence of 17.7% in commercial poultry farms [10]. Interestingly, the four randomly analysed *Salmonella* strains in our study showed susceptibility to all tested antibiotics. Although this finding could suggest a lower AMR burden in *Salmonella* from diseased chickens, the sample size was too low to enable valid prevalence estimation. Therefore, future studies must target larger numbers to account for rare phenotypes and genotypes.

In recent years, *Enterobacter* has emerged as the third *Enterobacteriaceae* showing resistance to third-generation cephalosporins (3GCs), after *E. coli* and *Klebsiella* [21]. This study revealed the presence of CTX resistance not only in *E. coli*, but *Enterobacter* as well. One out of eight (12.5%) *Enterobacter* and three out of six (50%) *E. coli* showed significantly high CTX MICs of at least 512 µg/mL. Globally, 3GCs are administered to the parent flock or day-old chicks, which contributes to increased levels of CTX resistance [27]. Additionally, CTX resistance could result from the selection pressure created by other antibiotics if ESBL genes coexist with other AMR genes on the same mobile genetic element [8]. Additionally, the administration of antibiotics through feed and water has contributed to increased levels of resistance, as this allows the uptake of antimicrobials by both infected and healthy chickens [28]. Such oral treatment regimens in chickens are prone to contamination during application by exposure to excreted faeces.

This is the first study to describe the presence of ESBL-encoding genes in diseased chickens in Zambia. Studies in healthy chickens in Zambia have reported a high prevalence of the *bla*_CTX-M_ gene among CTX-resistant *E. coli*. Accordingly, our study also revealed the presence of *bla*_CTX-M_ in all CTX-resistant *E. coli*, although none of the *Enterobacter* strains harboured this gene. Conversely, all *Enterobacter* isolates exhibited the *bla*_TEM_ gene. Interestingly, the *bla*_CTX-M_ and *bla*_TEM_ genes coexisted in two-thirds of the CTX-resistant *E. coli* isolates. This could be attributed to the presence of the genes on the same plasmid, as reported previously in Lusaka, Zambia [29], and in rural Nepal, where *E. coli* co-harboured the genes *bla*_CTX-M_ and *bla*_TEM_ [30]. Studies performed in Zambia reported a 13% prevalence of *bla*_CTX-M_ in *E. coli* isolated from market-ready chickens [2]. Another study performed on commercial poultry farms in Zambia’s Copperbelt Province revealed 12.8% occurrence of *bla*_CTX-M_ in *Salmonella* [10].

The detection *bla*_CTX-M_ genes in diseased chickens has public health implications, as the transmission of drug-resistant pathogenic strains to humans may cause severe, hard-to-treat infection. It is assumed that not only is poultry a zoonotic risk to humans, but poultry also acts as a reservoir for ESBL-producing bacteria. Several studies have suggested the transmission of *bla*_CTX-M_-producing *Enterobacteriaceae* between humans and animals through horizontal gene transfer and clonal expansion [31,32]. For instance, the *bla*_CTX-M_ gene has been detected on plasmids shared by human and poultry *E. coli* strains [33]. Additionally, similar *bla*_CTX-M_-positive *E. coli* strains have been found in humans and poultry, suggesting clonal dissemination. This transmission could be attributed to the poor handling of poultry in abattoirs or the increase in backyard poultry houses, where proper biosecurity maybe lacking.

The increase in the presence of *bla*_CTX-M_ could be attributed to the general increase in antimicrobial usage over recent years. In our study, amplicon sequencing of *bla*_CTX-M_ revealed the predominance of *bla*_CTX-M-15_ (5/6, 83%) among the CTX-resistant *E. coli* isolates. These findings are in line with the fact that *bla*_CTX-M-15_ is the most widely spread ESBL genotype globally [34]. The emergence of *bla*_CTX-M-15_ has been attributed to the clonal spread of the *E. coli* O25b:H4-ST131 pandemic clone. However, we did not perform multilocus sequence typing on our strains.

MDR bacteria pose significant danger to the public as common infections which were once easily treatable become fatal. This maybe because antibiotics that were once effective become useless and next-generation antibiotics are often expensive [35]. The *bla*_CTX-M-15_-positive *E. coli* isolates analysed in this study also portrayed an MDR phenotype which included tetracyclines and sulfonamides. The observed MDR could be attributed to the fact that, in Zambia, farmers are still using antibiotics to optimise their production. This is supported by antibiotic sales data which show that large amounts of antibiotics belonging to various classes are sold indiscriminately (Figure 3). Moreover, MDR can also be selected by only one antibiotic since AMR genes usually reside together on mobile genetic elements, allowing for simultaneous selection by a single drug [36].

Unlike *E. coli*, the clinical relevance of *Enterobacter* in poultry has not been well documented in Zambia, which could be attributed to the fact that *Enterobacter* rarely causes disease in immunocompetent chickens [21]. Data from this study has shown that *Enterobacter* does indeed carry the gene *bla*_TEM_, which may, in turn, be transmitted to humans. The MDR phenotype profile of the *bla*_TEM_-positive *Enterobacter* is alarming and calls for the urgent need for diagnostics before dispensing antimicrobials. This will allow for the more prudent use of antimicrobials and, in turn, limit the spread of AMR. *Enterobacter* rarely causes disease in immunocompetent chickens; thus, its presence probably goes unnoticed. Therefore, appropriate biosecurity measures in poultry houses would play a vital role in preventing its spread.

### Study Limitations

The study lacked MLST and whole-genome-based comparison analysis to assess the possibility of transmission between humans and poultry. In addition, this study only assessed a limited number of AMR genes. WGS would identify other AMR genes, mutations, serogroups, phylogroups, virulence genes, plasmids, and mobile genetic elements. The identification of various virulence factors would confirm the pathogenicity of the strains. Furthermore, our study only analysed 14 isolates, which may not have been enough to elucidate the association between CTX resistance and ESBL genes. The limited sample size may not fully represent the diversity of resistance patterns within the entire population of isolates. Therefore, future studies with a larger sample size will be required. Phylogenetics was not carried out, which hindered the study; we did not derive a clear picture of the genetic diversity and evolutionary aspects of these resistant strains.

## 4. Materials and Methods

### 4.1. Study Area and Sampling

**Figure 5:**
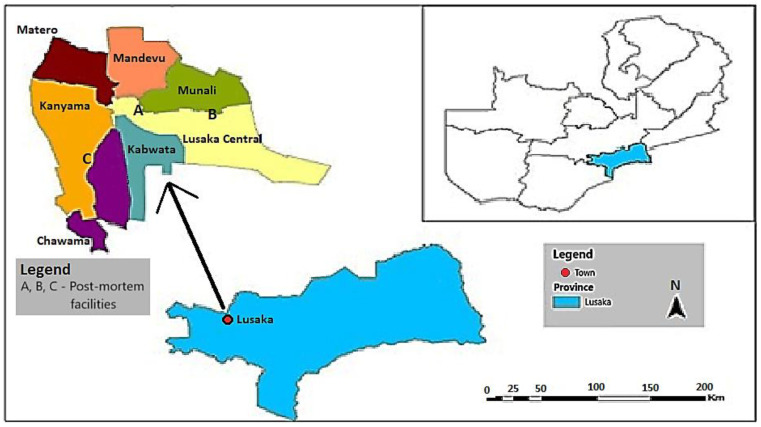
Map of Zambia showing the study areas.

The study was conducted in districts of Lusaka (Lusaka Central, Kanyama, Munali, Matero, Chawama, and Mandeveu areas) (Figure 5), the capital city of Zambia, which has a total population of 3,042,000 [1]. Between April 2021 and December 2021, a total of 215 samples from 91 diseased chickens were aseptically collected from three different post-mortem facilities in Lusaka.

### 4.2. Antimicrobial Usage Data

Antimicrobial usage (AMU) was estimated indirectly from antibiotic imports and sales recorded by one of Lusaka’s major suppliers of livestock antibiotics. The company sells antibiotics to retail agrovet shops, as well as directly to farmers on a prescription basis. The importation and sales data were used to provide information on AMU and guide the classes of antibiotics for use in susceptibility tests.

### 4.3. Bacterial Isolation

In total, 213 different internal organs (heart, liver, lung, and spleen) were collected from 91 diseased chickens aged between three and five weeks old. Post mortem, different criteria, such as pneumonia, pericarditis, congestion of the heart, congestion of the lungs, perihepatitis, necrosis, and bronzy liver, were used to collect the samples. The samples were placed on ice and transported to the lab.

A sterile loop was then used to swab the tissue sample and inoculated on the surface of the MacConkey agar (Oxoid LTD, Hampshire, UK), blood agar, and xylose lysine deoxycholate plates (Oxoid LTD, Hampshire, UK). The plates were incubated at 37 °C for 24 h followed by phenotypic identification using biochemical tests.

The biochemical tests were performed using the analytical profile index API^®^ Gram-negative microbial kit (bioMe’rieux, Midrand, South Africa). This was employed to identify *Enterobacteriaceae* to species level. *E. coli* MG1655 was used as the positive control.

### 4.4. Antibiotic Sensitivity Testing

Antimicrobial sensitivity was performed using the Kirby–Bauer disk diffusion method on Mueller–Hinton Agar (Becton, Dickinson and Company, Franklin Lakes, NJ, USA). The antibiotics disks (Becton, Dickinson and Company, Frankllin Lakes, NJ, USA) used included tetracycline (30 µg), ampicillin (10 µg), erythromycin (10 µg), cotrimoxazole (10 µg), amoxicillin (10 µg), gentamicin (10 µg), and penicillin (30 µg). All results were interpreted according to the Clinical and Laboratory Standards Institute (CSLI) guidelines [37].

### 4.5. Cefotaxime (CTX) Minimum Inhibitory Concentration (MIC)

The broth microdilution test was used to quantify CTX resistance [38]. Briefly, CTX resistance was confirmed by inoculating all 103 positive isolates identified in Table 2 on Luria–Bertani (LB) (Oxoid LTD, Hampshire, UK) agar plates supplemented with 1 µg/mL of CTX at 37 °C for 18 h. Of the 103 isolates, only *E. coli* and *Enterobacter* grew on the CTX-supplemented agar plates. Next, single colonies of *E. coli* and *Enterobacter* were picked from each plate and transferred to LB broth supplemented with 1 µg/mL CTX at 37 °C for 18 h, at 175 rpm. Finally, the cultures were diluted 10,000-fold and added to two-fold serial dilutions of CTX in a 96-well plate in triplicate. MIC was defined as the lowest CTX concentration that inhibited visible bacterial growth. *E. coli* strain MG1655 was used for quality control.

### 4.6. Detection of ESBL Genes by PCR

To screen for various ESBL genes (*bla*_CTX-M_, *bla*_TEM_, *bla*_SHV_, and *bla*_OXA_), the *E. coli* and *Enterobacter* isolates were subjected to PCR using primers in Table 4 which were sourced from the existing literature [39,40]. gDNA was extracted using the ZymoBIOMICS^®^ DNA Miniprep Kit, following the manufacturer’s instructions. PCR was performed using ExTaq HS (TaKaRa, Japan), with a total reaction volume of 50 µL consisting of 5 µL 10× ExTaq buffer, 4 µL of dNTP mixture, 2 µL of DNA template, 5 µL of both forward and reverse primers (10 µM each) (Table 2), 28.75 µL of nuclease-free water, and 0.25 µL of Takara Ex Taq HS. PCR conditions for *bla*_CTX-M_, *bla*_SHV_, and *bla*_OXA_ were denaturation at 98 °C for 2 min, followed by 25 cycles of template denaturation at 98 °C for 10 s, annealing at 60.5 °C for 5 s, and extension at 72 °C for 1 min with a final extension at 72 °C for 8 min. The PCR conditions for *bla*_TEM_ were 94 °C for 7 min, followed by 30 cycles of template denaturation at 94 °C for 30 s, primer annealing at 57 °C for 30 s and 72 °C for 5 min, whilst the final extension of 72 °C was for 5 min. PCR products were visualised under UV light after electrophoresis using 1.5% agarose gel.

### 4.7. PCR Product Purification and Cycle Sequencing

PCR products were purified using the MinElute PCR purification kit (QIAGEN, Hilden, Germany), according to the manufacturer’s instructions. The BigDye Terminator v3.1 kit (Applied Biosystems, Waltham, MA, USA) was then used for sequencing PCR, followed by the purification of excess buffers and unincorporated dNTPs using the ethanol precipitation method. Capillary electrophoresis was then performed using the ABI 3500 Genetic Analyzer (Applied Biosystems, Waltham, MA, USA).

### 4.8. Data Analysis

#### 4.8.1. AMR Data Analysis

Sales data were collected from the largest wholesale and retail supplier, inputted into an Excel sheet (Microsoft Excel, 2010), and cleaned. For antimicrobial susceptibility data, the inhibition zones were interpreted according to CSLI guidelines, and the data were manipulated using dplyr v1.0.7 [41] and reshape2 v1.4.4 [41] in Rstudio (Version 3). Finally, the data were displayed as tables and also visualised in ggplot2 v3.3.5 [42].

#### 4.8.2. ESBL Gene Sequence Analysis

The sequences were edited and assembled using the ATGC plug-in in Genetyx ver. 12 (GENETYX corporation, Tokyo, Japan). Sequences obtained in this study were then subjected to the BLAST analysis on the National Centre for Biotechnology Information (NCBI) website (http://www.ncbi.nlm.nih.gov/BLAST, accessed on 21 June 2023) for identity confirmation. The nucleotide sequences were then aligned with other nucleotide sequences obtained from GenBank using Clustal X2 [43]. Two of the sequences obtained were used as references and submitted to the National Centre for Biotechnology Institute. These were assigned accession numbers PP000852 and PP000853.

## 5. Conclusions

This is the first study in Zambia focusing on AMR patterns among *Enterobacteriaceae* strains isolated from diseased chickens. The study revealed a relationship between the estimated AMU and AMR, particularly with reference to tetracyclines and penicillins, highlighting the need to restrict unjustified access to antibiotics. Furthermore, MDR was identified among the isolates, and genotypic characterization identified *bla*_CTX-M_ and *bla*_TEM_ genes.

## Figures and Tables

**Table 1:** Species isolated at the three facilities.

Species	Facility			Total
	A	B	C	
*Enterobacter*	2	26	15	43
*E. coli*	5	11	4	20
*Salmonella*	2	8	0	10
*Klebsiella*	0	1	0	1
*Shigella*	0	5	3	8
*Yersinia*	0	2	0	2
*Citrobacter*	0	4	0	4
*Vibrio*	1	0	0	1
*Proteus*	4	5	1	10
*Morganella*	0	4	0	0
Total	14	66	23	103

**Table 2:** Sampling points for isolates for AST.

Facility		Pathogens		
	*Enterobacter*	*E. coli*	*Salmonella*	*Shigella*
A	3	3	3	1
B	1	1		1
C	1	1	1	

**Table 3:** CTX MICs for 14 *Enterobacteriaceae* strains.

SAMPLE ID	ORGANISM	CTX MIC	*bla*_TEM_	*bla*_CTX-M_
UZ 1	*E. coli*	≥512	+	+
UZ 2	*Enterobacter*	2	+	−
AV 1	*E. coli*	2	+	+
AV 2	*Enterobacter*	≥512	+	−
AV 3	*Enterobacter*	2	+	−
LS 1	*Enterobacter*	2	+	−
LS 2	*E. coli*	4	−	+
LS 3	*Enterobacter*	2	+	−
LS 4	*Enterobacter*	2	+	−
LS 5	*E. coli*	≥512	+	+
LS 6	*Enterobacter*	128	+	−
LS 7	*E. coli*	≥512	−	+
LS 8	*E. coli*	16	+	+
LS 9	*Enterobacter*	128	+	−

**Table 4:** Primers used in this study.

Primers	Target Gene	Sequence 5′–3′	Expected Amplicon Size	R Reference:
TEM1F TEM1R	*bla*_TEM_	ATGAGTATTCAACATTTCCG CTGACAGTTACCAATGCTTA	864	[39]
SHVF SHVR	*bla*_SHV_	GGTTATGCGTTATATTCGCC TTAGCGTTGCCAGTGCTC	865	[39]
CTX-MA1 CTX-MA2	*bla*_CTX-M_	*SCSATGTGCAG^≠^YACCAGTAA CCGC^¥^RATATGRTTGGTGGTG	544	[39]
OXAF OXAR	*bla_OXA_*	ATATCTCTACTGTTGCATCTCC AAACCCTTCAAACCATCC	619	[40]

Note: * S = G or C, ^≠^ Y = C or T, ^¥^ R = A or T.3.6 sequencing of PCR products [39,40].

## Data Availability

The data supporting the reported results are available on request from the corresponding author.
